# A high prevalence of multi-drug resistant Gram-negative bacilli in a Nepali tertiary care hospital and associated widespread distribution of Extended-Spectrum Beta-Lactamase (ESBL) and carbapenemase-encoding genes

**DOI:** 10.1186/s12941-020-00390-y

**Published:** 2020-10-21

**Authors:** Sulochana Manandhar, Raphael M. Zellweger, Nhukesh Maharjan, Sabina Dongol, Krishna G. Prajapati, Guy Thwaites, Buddha Basnyat, Sameer Mani Dixit, Stephen Baker, Abhilasha Karkey

**Affiliations:** 1 Patan Academy of Health Sciences, Oxford University Clinical Research Unit, Kathmandu, Nepal; 2 Centre for Tropical Medicine and Global Health, Nuffield Department of Medicine, University of Oxford, Oxford, UK; 3 Oxford University Clinical Research Unit, Ho Chi Minh City, Vietnam; 4 International Vaccine Institute, Seoul, South Korea; 5 Patan Academy of Health Sciences, Patan Hospital, Kathmandu, Nepal; 6 Center for Molecular Dynamics Nepal, Kathmandu, Nepal; 7 Cambridge Institute of Therapeutic Immunology & Infectious Disease (CITIID), Department of Medicine, University of Cambridge, Cambridge, UK

**Keywords:** Multi-drug resistance, Antimicrobial resistance, Nepal, ESBL, Gram-negative bacilli, Carbapenemase, ESKAPE

## Abstract

**Background:**

Multi-drug resistance (MDR) and extensive-drug resistance (XDR) associated with extended-spectrum beta-lactamases (ESBLs) and carbapenemases in Gram-negative bacteria are global public health concerns. Data on circulating antimicrobial resistance (AMR) genes in Gram-negative bacteria and their correlation with MDR and ESBL phenotypes from Nepal is scarce.

**Methods:**

A retrospective study was performed investigating the distribution of ESBL and carbapenemase genes and their potential association with ESBL and MDR phenotypes in *E. coli*, *Klebsiella* spp., *Enterobacter* spp. and *Acinetobacter* spp. isolated in a major tertiary hospital in Kathmandu, Nepal, between 2012 and 2018.

**Results:**

During this period, the hospital isolated 719 *E. coli*, 532 *Klebsiella* spp., 520 *Enterobacter* spp. and 382 *Acinetobacter* spp.; 1955/2153 (90.1%) of isolates were MDR and half (1080/2153) were ESBL producers. Upon PCR amplification, *bla*_TEM_ (1281/1771; 72%), *bla*_CTXM-1_ (930/1771; 53%) and *bla*_CTXM-8_ (419/1771; 24%) were the most prevalent ESBL genes in the enteric bacilli. *Bla*_OXA_ and *bla*_OXA-51_ were the most common *bla*_OXA_ family genes in the enteric bacilli (918/1771; 25%) and *Acinetobacter* spp. (218/382; 57%) respectively. Sixteen percent (342/2153) of all isolates and 20% (357/1771) of enteric bacilli harboured *bla*_NDM-1_ and *bla*_KPC_ carbapenemase genes respectively. Of enteric bacilli, *Enterobacter* spp. was the most frequently positive for *bla*_KPC_ gene (201/337; 60%). The presence of each *bla*_CTX-M_ and *bla*_OXA_ were significantly associated with non-susceptibility to third generation cephalosporins (OR 14.7, *p* < 0.001 and OR 2.3, *p* < 0.05, respectively).The presence of each *bla*_TEM_, *bla*_CTXM_ and *bla*_OXA_ family genes were significantly associated with ESBL positivity (OR 2.96, *p* < 0.001; OR 14.2, *p* < 0.001 and OR 1.3, *p* < 0.05 respectively) and being MDR (OR 1.96, *p* < 0.001; OR 5.9, *p* < 0.001 and OR 2.3, *p* < 0.001 respectively).

**Conclusions:**

This study documents an alarming level of AMR with high prevalence of MDR ESBL- and carbapenemase-positive ESKAPE microorganisms in our clinical setting. These data suggest a scenario where the clinical management of infected patients is increasingly difficult and requires the use of last-resort antimicrobials, which in turn is likely to intensify the magnitude of global AMR crisis.

## Background

Gram-negative bacilli, particularly those in the bacterial family Enterobacteriaceae (e.g. *Klebsiella* spp. and *Enterobacter* spp.) and *Acinetobacter* spp. are common causes of serious community and hospital-acquired infections. These Gram-negative bacilli are also members of the ESKAPE group of pathogens [1].These are notoriously associated with antimicrobial resistance (AMR) and frequently carry genes that induce resistance to three or more classes of antimicrobials, making them multi-drug resistant. Such multi-drug resistant Gram-negative bacilli represent a significant global public health problem as they are more commonly associated with worse outcomes than susceptible isolates [2–5].The situation with multi-drug resistant Gram-negative bacilli is particularly alarming in South Asia, which is considered as a global epicentre of these microorganisms [6, 7].

Extended-spectrum beta-lactamases (ESBLs) are enzymes that can hydrolyse and therefore inactivate beta-lactam antimicrobials such as penicillins, cephalosporins and monobactams. ESBL activity is an important mechanism by which Gram-negative bacilli exhibit resistance against beta-lactam antimicrobials. Multiple ESBL variants have been detected and grouped into several structural and evolutionary families which include TEM, SHV, CTX-M, PER, VEB, GES, BES, TLA and OXA [8]. Of these, CTX-M and TEM are the most common ESBLs, while OXA enzymes such as OXA-48, 23, 24, 51, 58 are common carbapenemases that are associated with Gram-negative bacteria that cause nosocomial infections [8, 9].

The possession of ESBLs increases the risk of treatment failure with beta-lactam antimicrobials, may contribute to the spread of AMR in Gram-negative bacteria and may complicate infection control in hospital [9, 10]. Therefore, detection and reporting for the presence of ESBLs in bacterial pathogens are important for clinical care. This is also essential for developing optimal infection control measures in hospital [10]. Modified disc diffusion tests are routinely used to detect the presence of ESBLs and establish the nature of ESBL phenotypes [11]. Though being useful, these phenotypic detection methods are labour intensive and do not determine the classes of genes that are associated with ESBL activity. Molecular methods like PCR, DNA hybridization, or whole genome sequencing (WGS) may be used alongside phenotypic test methods to detect the presence of ESBL and carbapenemase associated genes and generate additional information regarding the epidemiological distribution of key resistance genes in sentinel locations [10].

South Asia is a key location for antimicrobial resistant pathogens. A high prevalence of MDR and ESBL positive Enterobacteriaceae family bacteria and *Acinetobacter* have been reported across the region [12–19]. However, little is known about various ESBL and carbapenemase genes associated with these phenotypes in Gram-negative bacilli isolated in hospitals of Nepal. Furthermore, there are little data originating from this region on the association of these genotypes with the observed phenotypes. Such data are important for assessing the contribution of combined phenotypic and genetic tests on clinical care and providing better understanding of the impact of resistance genes on the epidemiology of circulating pathogens.

Here, aiming to better understand the epidemiology of multi-drug resistant microorganisms and improve clinical care in our healthcare facility, we determined the AMR profile of all *E. coli*, *Klebsiella* spp., *Enterobacter* spp. and *Acinetobacter* spp. isolated from in- and out-patients at a major tertiary hospital in Kathmandu, Nepal, between June 2012 and December 2018. We additionally screened them for the phenotypic presence of ESBL activity by disc diffusion method and performed PCR amplification to detect any ESBL and carbapenemase associated AMR genes. Lastly, we investigated the relationship between the presence of *bla*_CTX-M_, *bla*_TEM_ and *bla*_OXA_ genes with an ESBL or MDR phenotype. Understanding the association between AMR genotype and phenotype is crucial to assess the value of genetic detection methods in clinical settings.

## Methods

### Study design

This was a retrospective study of anonymised routine microbiology laboratory results originating from Patan Hospital in Lalitpur metropolitan city of the Kathmandu Valley, Nepal. All data regarding *E. coli*, *Klebsiella* spp., *Enterobacter* spp. and *Acinetobacter* spp. that were isolated from June 2012 to December 2018 were included in this study. These data were devoid of any personal identification information as this work was performed as a component of routine surveillance for infection control at Patan Hospital.

### Antimicrobial susceptibility testing

Antimicrobial susceptibility testing was performed at the time of bacterial isolation by modified Kirby-Bauer disc diffusion method, as previously described [20]. Zone size interpretations were performed following the appropriate Clinical and Laboratory Standards Institute (CLSI) guidelines [21]. The antimicrobials against which organisms were tested are listed in Additional file 1: Table S1; not all isolates were tested for all antimicrobials.

The bacteria producing resistant or intermediate response against tested antimicrobials were grouped as “non-susceptible” for the purposes of analysis. MDR was defined as an acquired non-susceptibility (without intrinsic resistance) to at least one agent of three or more antimicrobial classes. Intrinsic resistance was defined according to the CLSI guideline of 2014 [21]. The following intrinsic resistance were reported but ignored for the purpose of generating MDR profiles: (i) *Acinetobacter* spp. resistant against amoxicillin or penicillin, (ii) *E. coli*, *Klebsiella*, *Enterobacter,* or *Acinetobacter* spp. against vancomycin, teicoplanin, erythromycin or azithromycin. Phenotypic testing for ESBL positivity was conducted using the combination disc diffusion method with a beta-lactam antimicrobial disc alone and that in combination with a beta-lactamase inhibitor (clavulanic acid) [22]. The isolate was considered as ESBL positive if the zone of inhibition around the beta-lactamase inhibitor supplemented disc was ≥ 5 mm in comparison to the respective beta-lactam antimicrobial alone. The products of Mast diagnostics (Mast group Ltd., Liverpool, UK) namely D62C and D68C were used in this study.

### Detection of resistance genes

Bacterial DNA was extracted by suspending bacterial colonies in Phosphate buffered saline (PBS) and subjecting them to 100 °C for five minutes; the suspensions were centrifuged and the supernatant was used as template for PCR amplifications. The PCR amplifications were performed in multiplex following previously described primers and conditions. The ESBL targets were *bla*_CTXM-1_, *bla*_CTXM-2_, *bla*_CTXM-8_, *bla*_CTXM-9_, *bla*_CTXM-25_, *bla*_TEM_ and *bla*_SHV_ [23]. The carbapenem resistance genes tested were *bla*_OXA_, *bla*_KPC_, *bla*_OXA48_ [24], *bla*_NDM-1_ [25], *bla*_OXA1_4_30_, *bla*_OXA23_, *bla*_OXA24_, *bla*_OXA51_, *bla*_OXA58_ [26], *bla*_VIM_ and *bla*_IMP_ [27].

For analysis purpose, the detection of any of *bla*_OXA_, *bla*_OXA1_4_30_, *bla*_OXA23_, *bla*_OXA24_, *bla*_OXA48_, *bla*_OXA51_ or *bla*_OXA58_ was designated as *bla*_OXA_ positive. Similarly, the detection of any of *bla*_CTXM-1_, *bla*_CTXM-2_, *bla*_CTXM-8_, *bla*_CTXM-9_ or *bla*_CTXM-25_ was designated as *bla*_CTXM_ positive. Fisher’s exact test was used to test for an association between the detection of a resistance gene and a specific resistance phenotype (ESBL positive, MDR, or resistance to third and fourth generation cephalosporins). All analyses were performed using the statistical software R version 3.5.3 and R Studio version 1.0.143. Venn diagrams were generated using the UpSetR R package [28].

## Results

### Bacterial isolates

**Fig. 1:**
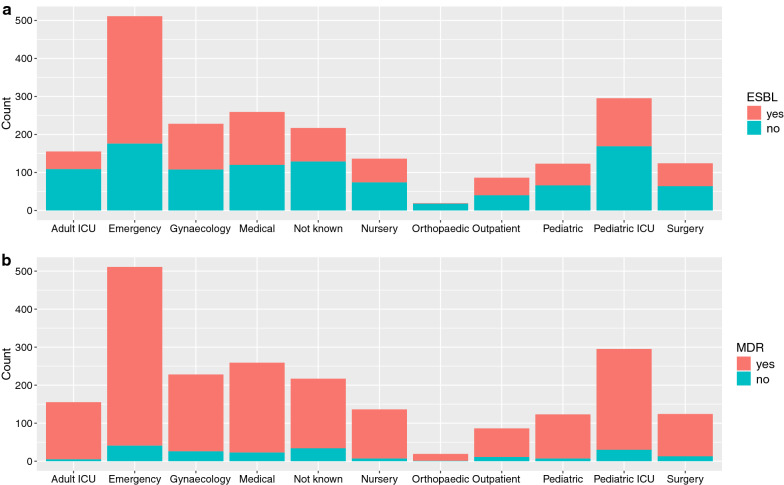
ESBL and MDR positivity status of isolates by ward. Count of ESBL-positive and -negative (**a**) and MDR and non-MDR isolates (**b**) stratified by hospital wards from where the samples were taken

Between June 2012 and December 2018, the microbiology laboratory at Patan hospital isolated 719 *E. coli*, 532 *Klebsiella* spp., 520 *Enterobacter* spp. and 383 *Acinetobacter* spp., totalling 2153 isolates (Table 1). A majority (512/2153; 23.8%) of isolates originated from the samples that were taken from the patients visiting emergency department followed by paediatric intensive care unit (PICU, 295/2153; 13.7%), gynaecology ward (287/2153; 13.3%) and general medical ward (208/2153; 9.7%). The distribution of ESBL-positivity and MDR in microorganisms by hospital departments is shown in Fig. 1. Half (1080/2153; 50.2%) of all bacterial isolates were ESBL positive (Table 2), which was predictably more common (> 50%) in *E. coli*, *Klebsiella* spp. and *Enterobacter* spp. isolates than in *Acinetobacter* spp. isolates (< 1%). Approximately 90% of *E. coli*, *Klebsiella* spp. and *Enterobacter* spp. were multi-drug resistant; this proportion was relatively lower in *Acinetobacter* spp. (82%).

**Table 1:** Summary of microorganisms

	n	%
Microorganisms (Total = 2153)		
* E. coli*	719	33.4
* Klebsiella* spp.	532	24.7
* Enterobacter* spp.	520	24.2
* Acinetobacter* spp.	382	17.7
Departments (Total = 2153)		
Emergency	512	23.8
Paediatric ICU	295	13.7
Gynaecology	287	13.3
Medical	208	9.7
Adult ICU	154	7.2
Nursery	136	6.3
Surgery	116	5.4
Paediatric	125	5.8
Not known	219	10.2
Outpatient	82	3.8
Orthopaedic	19	0.9
ESBL positive (Total = 2153)		
No	1073	49.8
Yes	1080	50.2

**Table 2:** ESBL positivity and MDR phenotype stratified by microorganisms

Microorganisms (N = 2153)	ESBL-neg		ESBL-pos		Non-MDR		MDR	
	n	%	n	%	n	%	n	%
* E. coli* (N = 719*)*	294	40.9	425	59.1	76	10.6	643	89.4
* Klebsiella* spp. (N = 532)	210	39.5	322	60.5	44	8.3	488	91.7
* Enterobacter* spp. (N = 520)	189	36.3	331	63.7	10	1.9	510	98.1
* Acinetobacter* spp. (N = 382)	380	99.5	2	0.5	68	17.8	314	82.2

### The distribution of AMR genes

The presence of screened AMR genes, stratified by the number of microorganisms tested is presented in Table 3. In *E. coli* isolates, 90.1% (648/719) were positive for *bla*_TEM_, 58.0% (417/719) were positive for *bla*_CTXM-8_ and 37.4% (269/719) were positive for *bla*_OXA_. In the *Enterobacter* spp., 56.2% (292/520) were positive for *bla*_OXA_, 59.6% (201/520) were positive for *bla*_KPC_, 80.3% (417/520) were positive for *bla*_CTXM-1_ and 57.9% (301/520) were positive for *bla*_TEM_. In *Klebsiella* spp., 77.4% (408/532) were positive for *bla*_CTXM-1_, 67.2% (357/532) were positive for *bla*_OXA_, 4.1% (22/532) were positive for *bla*_KPC_ and 62.5% (332/532) of tested isolates were positive for *bla*_TEM_. In *Acinetobacter* spp., 58.6% (218/382) were positive for *bla*_OXA51_, 36.0% (134/382) were positive for *bla*_OXA23_ and 20.7% (79/382) were positive for *bla*_NDM-1_.

**Table 3:** Summary of detection of AMR genes

Organisms	Genes	CTX-M1		CTX-M2		CTX-M8		CTX-M9		CTX-M25	
		n	%^a^	n	%^a^	n	%^a^	n	%^a^	n	%^a^
*E. coli*	neg	612	85.4	717	100	302	42.0	714	99.3	710	98.7
	pos	105	14.6	0	0	417	58.0	5	0.7	9	1.3
	n.t.^b^	2		2		0		0		0	
*Enterobacter*	neg	102	19.7	0	n.a	0	n.a	518	99.8	0	n.a
	pos	417	80.3	0	n.a	0	n.a	1	0.2	0	n.a
	n.t.^b^	1		520		520		1		520	
*Klebsiella*	neg	119	22.6	527	100	525	99.6	524	99.4	521	98.9
	pos	408	77.4	0	0	2	0.4	3	0.6	6	1.1
	n.t.^b^	5		5		5		5		5	
*Acinetobacter*	neg	368	98.9	372	100	372	100	372	100.0	372	100
	pos	4	1.1	0	0	0	0	0	0.0	0	0
	n.t.^b^	10		10		10		10		10	

Organisms	Genes	OXA		OXA1_4_30		OXA23		OXA24		OXA48		OXA51		OXA58	
		n	%^a^	n	%^a^	n	%^a^	n	%^a^	n	%^a^	n	%^a^	n	%^a^
*E. coli*	neg	450	62.6	0	n.a	0	n.a	0	n.a	715	99.4	0	n.a	0	n.a
	pos	269	37.4	0	n.a	0	n.a	0	n.a	4	0.6	0	n.a	0	n.a
	n.t.^b^	0		719		719		719		0		719		719	
*Enterobacter*	neg	228	43.8	0	n.a	0	n.a	0	n.a	221	65.6	0	n.a	0	n.a
	pos	292	56.2	0	n.a	0	n.a	0	n.a	116	34.4	0	n.a	0	n.a
	n.t.^b^	0		520		520		520				520		520	
*Klebsiella*	neg	174	32.8	0	n.a	0	n.a	0	n.a	475	89.5	0	n.a	0	n.a
	pos	357	67.2	0	n.a	0	n.a	0	n.a	56	10.5	0	n.a	0	n.a
	n.t.^b^	1		532		532		532		1		532		532	
*Acinetobacter*	neg	0	n.a	342	91.9	238	64.0	363	97.6	0	n.a	154	41.4	357	96.0
	pos	0	n.a	30	8.1	134	36.0	9	2.4	0	n.a	218	58.6	15	4.0
	n.t.^b^	382		10		10		10		382		10		10	

Organisms	Genes	IMP		KPC		NDM1		SHV		TEM		VIM	
		n	%^a^	n	%^a^	n	%^a^	n	%^a^	n	%^a^	n	%^a^
*E. coli*	neg	577	99.7	719	100	681	94.7	719	100	71	9.9	578	99.8
	pos	2	0.3	0	0.0	38	5.3	0		648	90.1	1	0.2
	n.t.^b^	140		0		0		0		0		140	
*Enterobacter*	neg	90	100	136	40.4	412	79.2	515	99.0	219	42.1	90	100
	pos	0	0	201	59.6	108	20.8	5	1.0	301	57.9	0	0.0
	n.t.^b^	183		183		0		0		0		430	
*Klebsiella*	neg	100	100	509	95.9	414	78.0	415	78.2	199	37.5	100	100
	pos	0	0	22	4.1	117	22.0	116	21.8	332	62.5	0	0.0
	n.t.^b^			1		1		1		1		432	
*Acinetobacter*	neg	0	n.a	0	n.a	303	79.3	361	97.0	300	78.5	0	n.a
	pos	0	n.a	0	n.a	79	20.7	11	3.0	82	21.5	0	n.a
	n.t.^b^	382		382		90		10		90		382	

^a^Percentage of positive or negative in all isolates tested for a particular gene

^b^Not tested

### The association of ESBL genes with ESBL phenotypes

For all subsequent analysis, isolates were classified as *bla*_OXA_ or *bla*_CTXM_ positive if any gene in the *bla*_OXA_ or *bla*_CTXM_ gene families were detected respectively. First, we assessed the association between phenotypic ESBL positivity and the presence of *bla*_OXA_, *bla*_CTXM,_ and *bla*_TEM_ (Table 4). The detection of each of these genes was significantly associated with an ESBL phenotype. The strength of association was variable between different classes with an odds ratio (OR) of 1.3 (95% CI 1.1–1.5, *p* < 0.05) for *bla*_OXA_, 2.96 (95% CI 2.5–3.6, *p* < 0.001) for *bla*_TEM_ and 14.2 (95% CI 11.2–18.1, *p* < 0.001) for *bla*_CTXM_ in Fisher’s test.

**Table 4:** Association between ESBL positivity, MDR or non-susceptibility to cephalosporins and presence of resistance genes

Genes	ESBL	non-ESBL	OR (95% CI)^c^	p-value^d^
*bla*_OXA_^a^				
OXA-neg	406	458		
OXA-pos	674	604	1.3 (1.1–1.5)	0.009
*bla*_CTXM_^b^				
CTXM-neg	112	660		
CTXM-pos	968	401	14.2 (11.2–18.1)	< 0.001
*bla*_TEM_				
TEM-neg	262	517		
TEM-pos	818	545	3.0 (2.5–3.6)	< 0.001

Genes	MDR	non-MDR	OR (95% CI)^c^	p-value^d^
*bla*_OXA_^a^				
OXA-neg	751	113		
OXA-pos	1198	80	2.3( 1.7–3.1)	< 0.001
*bla*_CTXM_^b^				
CTXM-neg	629	143		
CTXM-pos	1319	50	6.0 ( 4.2–8.6)	< 0.001
*bla*_TEM_				
TEM-neg	680	99		
TEM-pos	1269	94	2.0 ( 1.4–2.7)	< 0.001

Genes	Cephal. ¾ non-susceptible	Cephal. ¾ susceptible	OR (95% CI)^c^	p-value^d^
*bla*_OXA_^a^				
OXA-neg	825	35		
OXA-pos	1244	26	2.0 ( 1.2–3.5)	0.008
*bla*_CTXM_^b^				
CTXM-neg	710	54		
CTXM-pos	1358	7	14.7 ( 6.6–38.6)	< 0.001
*bla*_TEM_				
TEM-neg	746	27		
TEM-pos	1323	34	1.4 (0.8–2.4)	0.22

Cephal. 3/4, 3rd or 4th generation cephalosporin

^a^An isolate was classified as *bla*_OXA_ positive if *bla*_OXA1_4_30_, *bla*_OXA_, *bla*_OXA-23_, *bla*_OXA-24_, *bla*_OXA-48_, *bla*_OXA-51_ or *bla*_OXA-58_ was detected

^b^An isolate was classified as *bla*_CTXM_ positive if *bla*_CTXM-1_, *bla*_CTXM-2_, *bla*_CTXM-8_, *bla*_CTXM-9_ or *bla*_CTXM-25_ was detected

^c^Odds ratio (OR and 95% CI) of bacterial isolate to be ESBL positive, multi-drug resistant or resistant to 3rd or 4th generation cephalosporin in the presence of a respective gene (gene family)

^d^p-value from Fisher’s exact test

**Fig. 2:**
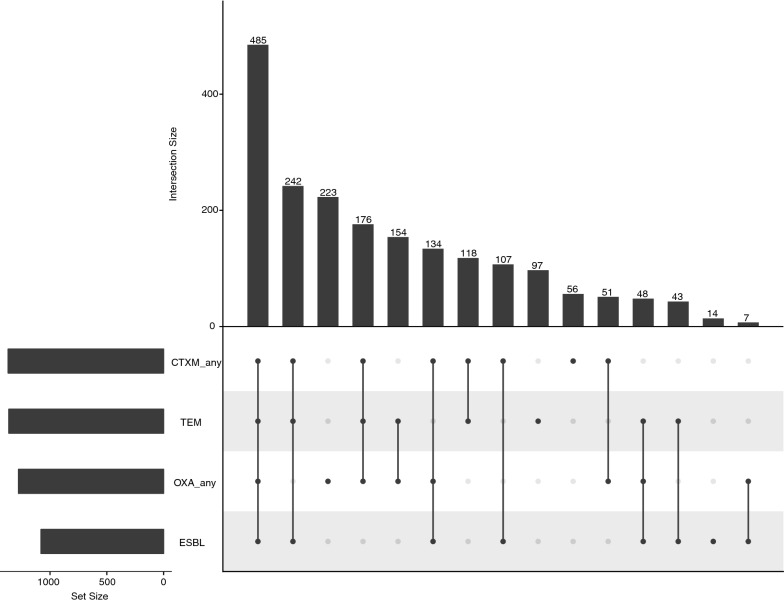
Association between ESBL positivity and combinations of resistance genes *bla*_OXA_, *bla*_CTXM_ and *bla*_TEM_. Black circles represent ESBL positive and/or the detection of resistance genes of *bla*_OXA_ family, *bla*_CTXM_ family or *bla*_TEM_. A grey circle indicates that a characteristic was not detected. The vertical bars indicate the number of isolates in the group summarized directly below. The horizontal bars indicate the total number of isolates positive for resistance genes of *bla*_OXA_ family, *bla*_CTXM_ family, *bla*_TEM_ or for ESBL positivity. An isolate was classified as *bla*_OXA_ positive if any gene of *bla*_OXA_ family was detected. The same is true for *bla*_CTXM_ genes. Only the isolates that were tested for ESBL positivity, at least one member of *bla*_OXA_ family, at least one member of *bla*_CTXM_ family and *bla*_TEM_ were included

Next, the association between ESBL positivity and various combinations of *bla*_OXA_, *bla*_CTXM_ and *bla*_TEM_ resistance genes were investigated. The isolates that were tested for at least one member of *bla*_OXA_ family, one member of *bla*_CTXM_ family and *bla*_TEM_ were analysed. A distribution of different combinations of *bla*_OXA_, *bla*_CTXM_ and *bla*_TEM_ resistance genes with ESBL positivity is shown in Fig. 2. The most frequent (485/1955; 24.8%) combination of genes that was associated with ESBL positivity was at least one member of *bla*_OXA_ family gene, with at least a member of *bla*_CTXM_ family gene and *bla*_TEM_ gene. A smaller proportion of isolates (176/1955; 9.0%) was PCR positive for *bla*_OXA_, *bla*_CTXM_ and *bla*_TEM_, but was phenotypically ESBL negative. The second most common (242/1955; 12.3%) gene combination associated with ESBL positivity was *bla*_CTXM_ with *bla*_TEM_. The proportion of isolates that were phenotypically ESBL positive, but PCR amplification negative for *bla*_OXA_, *bla*_CTXM_ and *bla*_TEM_ was 0.7% (14/1955).

### The association of ESBL genes with non-susceptibility to third and fourth generation cephalosporins

**Fig. 3:**
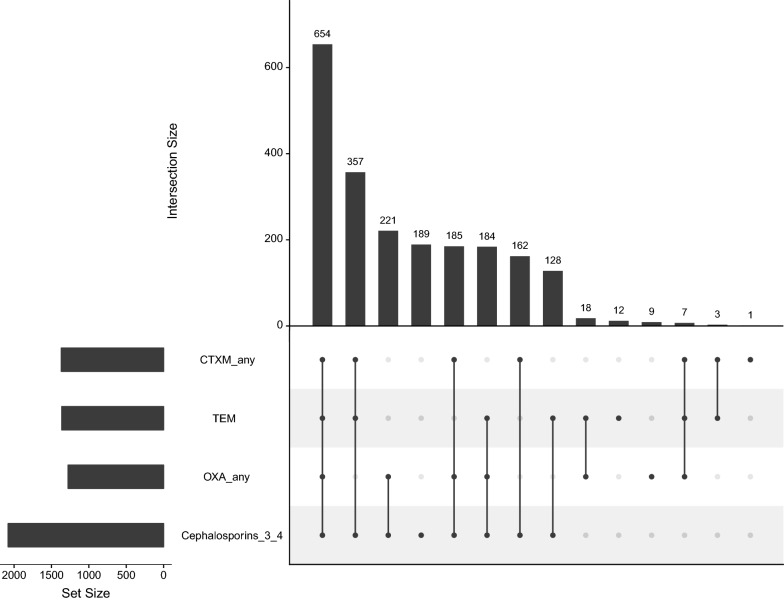
Association between non-susceptibility to 3rd and 4th generation cephalosporins and combinations of resistance genes *bla*_OXA_, *bla*_CTXM_ and *bla*_TEM_. Black circles represent non-susceptibility to cephalosporins (3rd/4th gen.) and/or the detection of resistance genes of *bla*_OXA_ family, *bla*_CTXM_ family or *bla*_TEM_. A grey circle indicates that a characteristic was not detected. The vertical bars indicate the number of isolates in the group summarized directly below. The horizontal bars indicate the total number of isolates positive for resistance genes of *bla*_OXA_ family, *bla*_CTXM_ family, for *bla*_TEM_ or for non-susceptibility to 3rd and 4th generation cephalosporins. An isolate was classified as *bla*_OXA_ positive if any gene of *bla*_OXA_ family was detected. The same is true for *bla*_CTXM_ genes. Only isolates that had a known 3rd and 4th generation cephalosporin resistance profile and were tested for at least one member of *bla*_OXA_ family, at least one member of *bla*_CTXM_ family and *bla*_TEM_ were included

We also tested for the association between non-susceptibility to third and fourth generation cephalosporins (Cefotaxime, Cefepime, Ceftriaxone and Cefixime) and the presence of *bla*_OXA_, *bla*_CTXM,_ or *bla*_TEM_ (Table 4). The *bla*_CTXM_ and *bla*_OXA_ gene families were significantly associated with non-susceptibility to third and fourth generation cephalosporins with odds ratios of 14.7 (95% CI 6.6–38.6, *p* < 0.001) and 2.03 (95% CI 1.2–3.5, *p* < 0.05) respectively. The most frequent combination (654/2121; 30.8%) associated with non-susceptibility against third and fourth generation cephalosporins was the presence of all three *bla*_OXA_, *bla*_CTXM_, and *bla*_TEM_ genes, followed by the presence of *bla*_CTXM_ and *bla*_TEM_ only (357/2121; 16.8%). Only 8.9% (189/2121) of isolates that were phenotypically non-susceptible to third and fourth generation cephalosporins were PCR amplification negative for *bla*_OXA_, *bla*_CTXM,_ and *bla*_TEM_ (Fig. 3).

### The association of ESBL genes with MDR

**Fig. 4:**
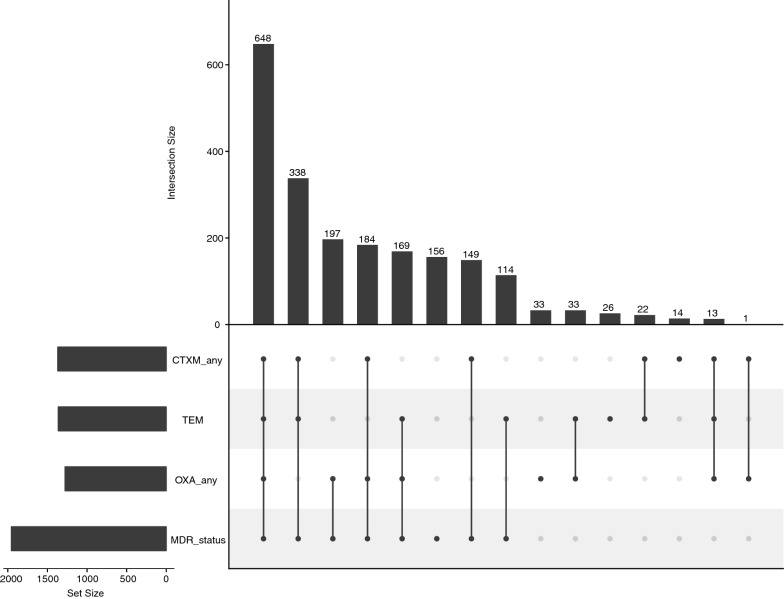
Association between MDR and combinations of resistance genes *bla*_OXA_, *bla*_CTXM_ and *bla*_TEM_. Black circles represent MDR and/or the detection of resistance genes of *bla*_OXA_ family, *bla*_CTXM_ family or *bla*_TEM_. A grey circle indicates that a characteristic was not detected. The vertical bars indicate the number of isolates in the group summarized directly below. The horizontal bars indicate the total number of isolates positive for resistance genes of *bla*_OXA_ family, *bla*_CTXM_ family, for *bla*_TEM_ or for MDR phenotype. An isolate was classified as *bla*_OXA_ positive if any gene of *bla*_OXA_ family was detected. The same is true for *bla*_CTXM_ genes. Only isolates that had an AMR profile and were tested for at least one member of *bla*_OXA_ family, at least one member of *bla*_CTXM_ family and *bla*_TEM_ were included

We similarly investigated for any potential association between the presence of *bla*_OXA_, *bla*_CTXM_ or *bla*_TEM_ genes with MDR (Table 4). A multi-drug resistant phenotype was significantly associated with the presence of *bla*_CTXM_ (OR 5.99, 95% CI 4.2–8.6, *p* < 0.001), *bla*_OXA_ (OR 2.3, 95% CI 1.7–3.1, *p* < 0.001) and *bla*_TEM_ (OR 1.96, 95% CI 1.4–2.7, *p* < 0.001). Additionally, the most common gene combinations associated with MDR were *bla*_OXA_ and *bla*_CTXM_ with *bla*_TEM_ genes (648/2097; 30.9%), followed by *bla*_CTXM_ with *bla*_TEM_ (338/2097; 16.1%). A small fraction of isolates (156/2097; 7.4%) were PCR amplification negative for *bla*_OXA_, *bla*_CTXM_ and *bla*_TEM,_ but were multi-drug resistant phenotypes (Fig. 4).

**Fig. 5:**
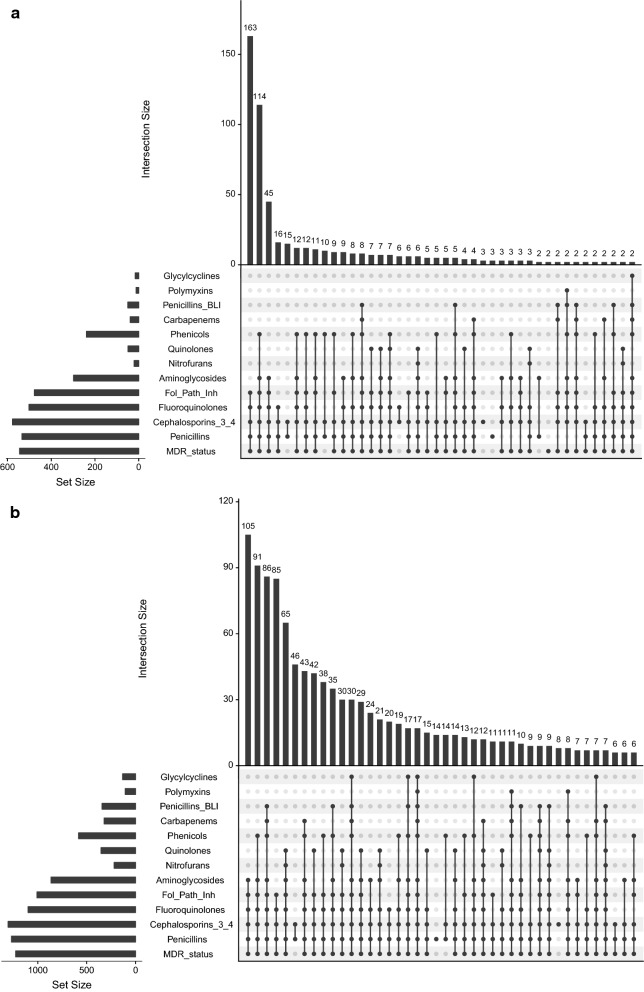
AMR patterns for outpatients and inpatients. AMR patterns are presented for outpatients (emergency and outpatients departments) in (**a**) and for inpatients (all other hospital wards) in (**b**). Only the 42 most frequent combinations are shown. Black circles represent resistance to the antimicrobials groups and/or multidrug resistance (MDR). The vertical bars represent the number of isolates characterized by the AMR pattern described under the bar. The horizontal bars indicate the total number of isolates resistant to the different groups, or MDR

As MDR was defined as an acquired non-susceptibility to at least one agent in three different classes of antimicrobials, a multi-drug resistant phenotype can originate from a multitude of individual antimicrobial resistant phenotypes. Therefore, we compared the pattern of resistance for the isolates originating from outpatients (emergency ward and outpatient department, Fig. 5a) and that from inpatients (all other wards, Fig. 5b). For the microorganisms originating from the outpatients, the three most frequent AMR combinations accounted for over half (322/535; 60.2%) of all outpatient isolates. For the microorganisms originating from the inpatients, the three most frequent AMR combinations accounted only for 26% of all inpatient isolates (282/1057) and the diversity of resistant phenotypes was much broader than that from outpatient isolates.

## Discussion

In this study, very high prevalence of ESBL positivity and MDR was found in Enterobacteriaceae family bacteria (*E. coli*, *Klebsiella* spp. and *Enterobacter* spp.). Similar MDR prevalence has been reported in other studies from Nepal, but the ESBL positive prevalence we observed (over 50%) is higher than others have reported. For example, one study from Nepal found that in community-acquired infections caused by *E. coli*, 24% were ESBL positive and 78% had MDR [12]. Another hospital-based study from Nepal showed that in urine samples, 27% of *E. coli* were ESBL positive and 97% of Enterobacteriaceae bacteria (mostly *E. coli* and *Klebsiella* spp.) were multi-drug resistant [13]. For *Acinetobacter* spp., we report over 81% MDR, similar to what others have reported in ICUs in Nepal, but the ESBL prevalence we found (< 1% in *Acinetobacter* spp.) is much lower than what has been reported (around 12–13%) [15, 16, 29]. In the context of South Asia, 79% of *Acinetobacter* spp., 71% of *Klebsiella* spp. and 54% of *E. coli* isolated from young infants were estimated to be multi-drug resistant strains [19]. ESBL positivity was confirmed in 42% of *K. pneumoniae* and 33% of *E. coli* isolated from several hospitals in India [30], 40% in Enterobacteriaceae isolates from Pakistan [18] and 16% in enteric and non-enteric Gram-negative bacilli from Bangladesh [31].

To limit the spread of antimicrobial resistant microorganisms, to guide clinical care and improve patients’ outcome, it is essential to have precise knowledge on AMR profile of the infecting bacteria prior to starting antimicrobial therapy to select an appropriate drug. Antimicrobial sensitivity testing and detection of ESBL positivity can be performed by classical phenotypical methods such as disc diffusion, which is labour intensive and time consuming. Automated systems exist, but their installation, running and maintenance costs are often a barrier in low resource settings. Alternatively, simple genetic methods such as PCR can detect AMR genes rapidly and with high sensitivity [2, 10, 32]. A better understanding of the association between phenotype and genotype is a prerequisite to evaluate the usefulness of genetic detection methods in clinical settings. In this study, we assessed the correlation between the detection of *bla*_CTXM_, *bla*_TEM_ and *bla*_OXA_ genes by PCR and ESBL positivity or MDR as detected phenotypically by disc diffusion.

For *E. coli*, *Enterobacter* spp. and *Klebsiella* spp., the resistance genes most frequently detected were *bla*_OXA_, *bla*_TEM_, *bla*_CTXM-1_ and *bla*_CTXM-8_.This is consistent with a study performed in 2012–2013 at Kathmandu Medical College and Teaching Hospital, in which *bla*_CTXM_ and *bla*_TEM_ were frequently detected in ESBL positive *E. coli* [14]. A study from Bangladesh reported *bla*_CTXM-1_ (51%) and *bla*_SHV_ (27%) to be the most common ESBL genes in *Klebsiella pneumoniae* [17]*.* In Pakistan and India, as in our study, *bla*_OXA_, *bla*_TEM_ and *bla*_CTXM_, in addition to *bla*_SHV_ were detected to be common AMR genes in Enterobacteriaceae isolates [18, 30]. For *Acinetobacter* spp., *bla*_OXA-51_, *bla*_OXA-23_ and *bla*_NDM-1_were the most frequently detected AMR genes, as found by others in Nepal, India and Bangladesh [33–35].

The detection of any gene of the *bla*_CTXM_ family was strongly associated with ESBL positivity (OR 14.2, 95% CI11.2–18.1), MDR (OR 5.99, 95% CI 4.2–8.6) and resistance to 3rd and 4th generation cephalosporins (OR 14.7, 95% CI 6.6–38.6). The detection of *bla*_TEM_ was strongly associated with ESBL positivity (OR 2.96, 95% CI 2.5–3.6) and MDR (OR 1.96, 95% CI 1.4–2.7). An association between *bla*_TEM_ and resistance to 3rd and 4th generation cephalosporins was suggested by an OR of 1.4, but it was not statistically significant (95% CI 0.8–2.4), perhaps due to low number of isolates susceptible to 3rd and 4th generation cephalosporins. Finally, the detection of any gene of the *bla*_OXA_ family was associated with multi-drug resistant phenotype (OR 2.3, 95% CI 1.7–3.1). This suggests that the detection of *bla*_TEM_, or any gene of the *bla*_CTXM_ or *bla*_OXA_ family is an important index for multi-drug resistant phenotype, and as expected, the detection of a *bla*_CTXM_ family gene or *bla*_TEM_ indicates a higher odds of ESBL positive phenotype. Collectively, this suggests that the detection of key AMR genes by molecular methods is an important index for ESBL positivity and MDR in bacterial isolates.

In this study, we also described different AMR patterns found in the isolates originating from emergency and outpatient wards (outpatients) compared to all other wards of the hospital (inpatients), which may reflect different usage of antimicrobials in community compared to the hospital. It can be assumed that isolates originating from outpatients reflect what is circulating in communities. For outpatients, the three most frequent AMR combinations accounted for more than half of all isolates. Perhaps people contracting community-acquired infections predominantly self-medicate, which results in exposure to the limited array of antimicrobials that are freely available in shops and pharmacies. Only infections that are (or have become) resistant to those drugs prompt a visit to the hospital. As a result, infections seen in outpatients have similar resistance profile. In contrast, a higher diversity of AMR combinations found in inpatients may reflect a wider array of antimicrobials available and used at hospitals.

## Conclusions

MDR along with possession of ESBL and carbapenemase associated resistance genes among Gram-negative bacilli pose a serious problem in therapeutic management of patients. The compromised infection control and inadequate antimicrobial usage policies coupled with high burden of Gram-negative bacilli possessing transferable antimicrobial resistance genes in resource limited settings set out an ideal scenario for the emergence and dissemination of multi-drug resistant pathogens. In this extensive retrospective study, a high burden of multi-drug resistant clinical Gram-negative bacilli possessing diverse ESBL and carbapenemase resistance *bla* genes were evidenced. Further, our study signifies that there is a high probability of Gram-negative bacilli to be multi-drug resistant and ESBL positive in case of detection of any of *bla*_TEM_, *bla*_CTXM_ or *bla*_OXA_ family genes. Additionally, the detection of any of the *bla*_CTXM_ family genes highly implies that the Gram-negative bacilli are non-susceptible to the extended-spectrum beta-lactam antimicrobials.

## Supplementary information

**Additional file 1: Table S1.** Classes of antimicrobials and their members.

## Data Availability

All data generated or analysed during this study are included in this published article and its supplementary information files.

## Abbreviations

AMR: Antimicrobial resistance
CI: Confidence interval
CLSI: Clinical and laboratory standards institute
DNA: Deoxy-ribonucleic acid
ESBL: Extended-spectrum beta-lactamase
MDR: Multi-drug resistance
OR: Odds ratio
PCR: Polymerase chain reaction
PICU: Pediatric intensive care unit
WGS: Whole genome sequencing
XDR: Extensive-drug resistance

## References

[1] Rice LB (2008). Federal funding for the study of antimicrobial resistance in nosocomial pathogens: no ESKAPE. J Infect Dis.

[2] Exner M, Bhattacharya S, Christiansen B, Gebel J, Goroncy-Bermes P, Hartemann P (2017). Antibiotic resistance: What is so special about multidrug-resistant Gram-negative bacteria?. GMS Hyg Infect Control..

[3] World Health Organisation (2014). Global report on surveillance: antimicrobial resistance. World Heal Organ..

[4] Livermore DM (2012). Current epidemiology and growing resistance of gram-negative pathogens. Korean J Intern Med.

[5] Doi Y, Bonomo RA, Hooper DC, Kaye KS, Johnson JR, Clancy CJ (2017). Gram-negative bacterial infections: research priorities, accomplishments, and future directions of the antibacterial resistance leadership group. Clin Infect Dis..

[6] Laxminarayan R, Chaudhury RR (2016). Antibiotic resistance in india: drivers and opportunities for action. PLOS Med.

[7] Bhatia R, Narain JP (2010). The growing challenge of antimicrobial resistance in the South-East Asia Region–are we losing the battle?. Indian J Med Res.

[8] Paterson DL, Bonomo RA (2005). Extended-spectrum β-lactamases: a clinical update. Clin Microbiol Rev..

[9] Mathers AJ, Peirano G, Pitout JDD (2015). The role of epidemic resistance plasmids and international high-risk clones in the spread of multidrug-resistant Enterobacteriaceae. Clin Microbiol Rev.

[10] Pitout JDD, Laupland KB (2008). Extended-spectrum beta-lactamase-producing Enterobacteriaceae: an emerging public-health concern. Lancet Infect Dis.

[11] Bradford PA (2001). Extended-spectrum β-lactamases in the 21st century: characterization, epidemiology, and detection of this important resistance threat. Clin Microbiol Rev.

[12] Ansari S, Hari P, Gautam R, Shrestha S, Neopane P, Rimal B, Mandal F, Ansari SR, Chapagain ML, Ansari SRS, Nepal HP, Gautam R, Shrestha S, Neopane P (2015). Community acquired multi-drug resistant clinical isolates of *Escherichia coli* in a tertiary care center of Nepal. Antimicrob Resist Infect Control..

[13] Yadav KK, Adhikari N, Khadka R, Pant AD, Shah B (2015). Multidrug resistant Enterobacteriaceae and extended-spectrum β-lactamase producing *Escherichia coli*: a cross-sectional study in National Kidney Center. Nepal Antimicrob Resist Infect Control.

[14] Pokhrel R, Thapa B, Kafle R, Shah P, Tribuddharat C (2014). Co-existence of beta-lactamases in clinical isolates of *Escherichia coli* from Kathmandu. Nepal BMC Res Notes.

[15] Khanal S, Joshi DR, Bhatta DR, Devkota U, Pokhrel BM (2013). *β*-Lactamase-producing multidrug-resistant bacterial pathogens from tracheal aspirates of intensive care unit patients at national institute of neurological and allied sciences. Nepal ISRN Microbiol.

[16] Bhandari P, Thapa G, Pokhrel BM, Bhatta DR, Devkota U (2015). Nosocomial isolates and their drug resistant pattern in icu patients at national institute of neurological and allied sciences. Nepal Int J Microbiol.

[17] Khan ER, Aung MS, Paul SK, Ahmed S, Haque N, Ahamed F (2018). Prevalence and molecular epidemiology of clinical isolates of escherichia coli and klebsiella pneumoniae harboring extended-spectrum beta-lactamase and carbapenemase genes in Bangladesh. Microb Drug Resist.

[18] Abrar S, Hussain S, Khan RA, Ul Ain N, Haider H, Riaz S (2018). Prevalence of extended-spectrum-β-lactamase-producing Enterobacteriaceae: first systematic meta-analysis report from Pakistan. Antimicrob Resist Infect Control.

[19] Chaurasia S, Sivanandan S, Agarwal R, Ellis S, Sharland M, Sankar MJ (2019). Neonatal sepsis in South Asia: huge burden and spiralling antimicrobial resistance. BMJ..

[20] Bauer A, Kirby W, Sherris J, Turck M (1966). Antibiotic susceptibility testing by a standardized single disk method. Am J Clin Pathol.

[21] Clinical and Laboratory Standards Institute (2014). Standards for antimicrobial susceptibility testing; twenty-fourth informational supplement, CLSI document M100–S24.

[22] Thomson KS, Sanders CC (1992). Detection of extended-spectrum β-lactamases in members of the family Enterobacteriaceae: comparison of the double-disk and three-dimensional tests. Antimicrob Agents Chemother.

[23] Woodford N, Fagan EJ, Ellington MJ (2006). Multiplex PCR for rapid detection of genes encoding CTX-M extended-spectrum β-lactamases. J Antimicrob Chemother.

[24] Poirel L, Walsh TR, Cuvillier V, Nordmann P (2011). Multiplex PCR for detection of acquired carbapenemase genes. Diagn Microbiol Infect Dis.

[25] Nordmann P, Poirel L, Carrër A, Toleman MA, Walsh TR (2011). How to detect NDM-1 producers. J Clin Microbiol.

[26] Woodford N, Ellington MJ, Coelho JM, Turton JF, Ward ME, Brown S (2006). Multiplex PCR for genes encoding prevalent OXA carbapenemases in *Acinetobacter* spp. Int J Antimicrob Agents.

[27] Shirani K, Ataei B, Roshandel F (2016). Antibiotic resistance pattern and evaluation of metallo-beta lactamase genes (VIM and IMP) in *Pseudomonas aeruginosa* strains producing MBL enzyme, isolated from patients with secondary immunodeficiency. Adv Biomed Res.

[28] Lex A, Gehlenborg N, Strobelt H, Vuillemot R, Pfister H (2014). UpSet: visualization of intersecting sets. IEEE Trans Vis Comput Graph.

[29] Mishra S, Rijal B, Pokhrel B (2013). Emerging threat of multidrug resistant bugs—*Acinetobacter calcoaceticus* baumannii complex and Methicillin resistant *Staphylococcus aureus*. BMC Res Notes.

[30] Gautam V, Thakur A, Sharma M, Singh A, Bansal S, Sharma A (2019). Molecular characterization of extended-spectrum β-lactamases among clinical isolates of *Escherichia coli* & Klebsiella pneumoniae: a multi-centric study from tertiary care hospitals in India. Indian J Med Res.

[31] Jobayer M, Afroz Z, Nahar S, Begum A, Begum S, Shamsuzzaman S (2017). Antimicrobial susceptibility pattern of extended-spectrum beta-lactamases producing organisms isolated in a Tertiary Care Hospital, Bangladesh. Int J Appl Basic Med Res.

[32] Licker M, Anghel A, Moldovan R, Hogea E, Muntean D, Horhat F (2015). Genotype-phenotype correlation in multiresistant *Escherichia coli* and *Klebsiella pneumoniae* strains isolated in Western Romania. Eur Rev Med Pharmacol Sci.

[33] Joshi PR, Acharya M, Kakshapati T, Leungtongkam U, Thummeepak R, Sitthisak S (2017). Co-existence of bla OXA-23 and bla NDM-1 genes of *Acinetobacter baumannii* isolated from Nepal: antimicrobial resistance and clinical significance. Antimicrob Resist Infect Control.

[34] Vijayakumar S, Mathur P, Kapil A, Das BK, Ray P, Gautam V (2019). Molecular characterization & epidemiology of carbapenem-resistant Acinetobacter baumannii collected across India. Indian J Med Res.

[35] Khatun MN, Farzana R, Lopes BS, Shamsuzzaman SM (2015). Molecular characterization and resistance profile of nosocomial *Acinetobacter baumannii* intensive care unit of tertiary care hospital in Bangladesh. Bangladesh Med Res Counc Bull.

