# Supplementary material for: A high prevalence of multi-drug resistant Gram-negative bacilli in a Nepali tertiary care hospital and associated widespread distribution of Extended-Spectrum Beta-Lactamase (ESBL) and carbapenemase-encoding genes
Source: Ann Clin Microbiol Antimicrob. 2020 Oct 21;19:48. doi: 10.1186/s12941-020-00390-y (PMC7576804; doi:10.1186/s12941-020-00390-y)
Supplement: Supplementary file 1 — Additional file 1: Table S1. Classes of antimicrobials and their members. [file 12941_2020_390_MOESM1_ESM.docx]

**Additional material**

**Table S1 Classes of antimicrobials and their members**

| **Classes** | **Members** |
| --- | --- |
| Penicillin | Amoxicillin (AMX), Penicillin (PEN), Oxacillin (OXA), Ticarcillin (TIC) |
| 3^rd^ , 4^th^ generation cephalosporins | Cefotaxime (CTX), Cefepime (FEP), Ceftriaxone (CRO),Cefixime(CFM) |
| Fluoroquinolones | Ciproxine (CIP), Ofloxacin (OFX), Gatifloxacin (GAT), Levofloxacin (LVX) |
| Folate pathway inhibitors | Trimethoprim-sulphamethoxazole (SXT) |
| Aminoglycosides | Gentamicin (GEN), Amikacin (AMK), Tobramycin (TOB) |
| Nitrofurans | Nitrofurantoin (NIT) |
| Quinolones | Nalidixic acid (NAL) |
| Phenicols | Chloramphenicol (CHL) |
| Carbapenems | Meropenem (MRP), Imipenem (IMP) |
| Penicillins + beta-lactamase inhibitor | Ampicillin-sulbactam (AMP_SUL), Piperacillin-Tazobactam(PTZ) |
| Polymyxins | Colistin (CST) |
| Glycylcyclines | Tigecycline (TGC) |
| Monobactam | Aztreonam (ATM) |
| Macrolides | Erythromycin (ERY),Azithromycin(AZM) |
| Glycopeptides | Vancomycin (VAN), Teicoplanin (TEC) |
| 3^rd^/ 4^th^ generation cephalosporins + beta-lactamase inhibitor | Cefixime-sulbactam (CFM_SUL), Ceftazidime-sulbactam (CAZ_SUL), Ceftriaxone-sulbactam(CRO_SUL) |
